# Peripheral perfusion index in well newborns at 6 to 72 h of life at different altitudes: a multi-center study in China

**DOI:** 10.1007/s00431-022-04725-z

**Published:** 2022-12-16

**Authors:** Wei Hua, Conway Niu, Yan Xuan, Qu-ming Zhao, Yan Ren, Xue Hu, Zhi-xiu Wang, Jin-qiao Sun, Gesang Yangjin, Yalan Dou, Wei-li Yan, Xiao-jing Hu, Guo-ying Huang

**Affiliations:** 1 Children’s Hospital of Fudan University, Shanghai, China; 2 King Edward Memorial Hospital, Subiaco, WA Australia; 3 Hainan Women and Children Medical Center, Haikou, China; 4 People’s Hospital of Luchun County, Yunnan Province, China; 5 People’s Hospital of Xinjiang Uygur Autonomous Region, Ürümqi, China; 6 Yan’an Affiliated Hospital of Kunming Medical University, Kunming, China; 7 People’s Hospital of Golog Tibetan Autonomous Prefecture, Dawu Town, China; 8 Tibet Autonomous Region People’s Hospital, Lhasa, China; 9 Shanghai Key Laboratory of Birth Defects, Shanghai, China; 10 National Management Office of Neonatal Screening Project for CHD, Shanghai, China; 11 Pediatric Heart Center, Children’s Hospital of Fudan University, 399 Wan Yuan Road, Shanghai, 201102 People’s Republic of China

**Keywords:** Newborn, Altitude, Peripheral perfusion index, LMS

## Abstract

The purpose of this study is to obtain the reference range of peripheral perfusion index (PPI) of asymptomatic well newborns at 6 to 72 h of life at different altitudes. A population-based prospective cohort study was conducted in cities at different altitudes in China. Asymptomatic well newborns were enrolled consecutively from six hospitals with an altitude of 4 to 4200 m between February 1, 2020, and April 15, 2021. PPI was measured at 6, 12, 24, 48, and 72 h after birth on the right hand (pre-ductal) and either foot (post-ductal) using a Masimo SET Radical-7 oximeter. Fiftieth percentile reference curves of the pre- and post-ductal PPI values at 6–72 h after birth were generated using the Lambda Mu Sigma method. Linear mixed-effects regression was performed to determine the influence of different altitude levels on PPI values over different measurement time points. A total of 4257 asymptomatic well newborns were recruited for analysis. The median and quartile pre- and post-ductal PPI values at 6–72 h of life at different altitudes were 1.70 (1.20, 2.60) and 1.70 (1.10, 2.70) for all infants, 1.30 (1.10, 1.90) and 1.10 (0.88, 1.80) for infants at low altitude, 1.40 (1.00, 2.00) and 1.30 (0.99, 2.00) at mild altitudes, 1.90 (1.30, 2.50) and 1.80 (1.20, 2.70) at moderate altitudes, 1.80 (1.40, 3.50) and 2.20 (1.60, 4.30) for high altitudes, 3.20 (2.70, 3.70), and 3.10 (2.10, 3.30) for higher altitudes, respectively. Overall, both pre- and post-ductal PPI increased with altitude. The 50th percentile curves of pre- and post-ductal PPI values in well newborns at mild, low, moderate, and high altitudes were relatively similar, while the difference between the PPI curves of infants at higher altitudes and other altitudes was significantly different.

*  Conclusions*: With the increase of altitude, pre- and post-ductal PPI of newborns increases. Our study obtained the PPI reference values of asymptomatic well newborns at 6 to 72 h after birth at different altitudes from 4 to ≥ 4000 m.

**Table:** 

**What is Known:** *• Monitoring hemodynamics is very important to neonates. As an accurate and reliable hemodynamic monitoring index, PPI can detect irreversible damage caused by insufficient tissue perfusion and oxygenation early, directly, noninvasively, and continuously.*	
**What is New:** *• Our study obtained the PPI reference values of asymptomatic well newborns at 6 to 72 h after birth at different altitudes from 4 to ≥ 4000 m. With the increase of altitude, pre- and post-ductal PPI of newborns increase with statistical significance. Therefore, the values and disease thresholds of PPI for asymptomatic neonates should be modified according to altitudes.*

**What is Known:**

*• Monitoring hemodynamics is very important to neonates. As an accurate and reliable hemodynamic monitoring index, PPI can detect irreversible damage caused by insufficient tissue perfusion and oxygenation early, directly, noninvasively, and continuously.*

**What is New:**

*• Our study obtained the PPI reference values of asymptomatic well newborns at 6 to 72 h after birth at different altitudes from 4 to ≥ 4000 m. With the increase of altitude, pre- and post-ductal PPI of newborns increase with statistical significance. Therefore, the values and disease thresholds of PPI for asymptomatic neonates should be modified according to altitudes.*

## Introduction

Peripheral perfusion index (PPI) is a measurement index made available with new-generation non-invasive oximeters. The probes equipped with the new pulse oximeters release near-infrared light at 940 nm wavelength, which can be absorbed by hemoglobin. At the site in which monitoring is carried out, both pulsatile tissue (arterial blood flow) and non-pulsatile tissue (venous blood, muscle, and other tissues) absorb the emitted light. The amount of light absorbed by the non-pulsatile tissue is constant. PPI is the ratio of the amount of light absorbed by pulsatile tissue to that absorbed by non-pulsatile tissue. It is a relative evaluation of arterial perfusion and pulse intensity at the monitoring site [1, 2]. Light absorption rates are typically affected by changes in perfusion, and thus, PPI can quantify this change in real-time [2, 3]. 

Blood is pumped from the heart into the peripheral microcirculation through blood vessels, and as such, cardiac output and vasoconstriction are the main factors affecting PPI. When vasomotor tone remains constant in a single respiratory cycle, the measurement of PPI is mainly affected by blood volume at the monitoring site. When there is no significant change in hemoglobin, PPI values will change with the fluctuation of blood flow to the skin. Under normal circumstances, the skin perfusion in neonates is higher than its demand for oxygen. In pathological states, peripheral vascular resistance increases, and blood is preferentially distributed to the brain, heart, adrenal glands, and other key organs to provide essential oxygenation [1]. As a real-time, non-invasive, and continuous monitoring technology, the lower the PPI value, the lower the systemic perfusion [1]. Monitoring the PPI value of an infant is of great value in assessing his or her clinical condition [4]. Some studies have observed the reference range of PPI in infants [5–11], while some have gone on to explore the relationship between PPI and neonatal diseases [7, 12–24]. Some studies have even demonstrated that PPI can be used as a predictor of neonatal disease severity [11, 25–27]. In addition, PPI has potential important value in screening critical congenital heart disease (CCHD) in neonates. The study found that 0.7 may be an important threshold for the detection of left heart obstructive disease (LHOD) [10, 28–30]. The above studies have made a strong case for the significant value of PPI in neonatal monitoring, but these studies did not focus on the impact of altitude on PPI. We hypothesized that the PPI of asymptomatic well newborns at different altitudes is different and that findings from PPI studies at sea level, including the thresholds for predicting disease severity, are not directly applicable to neonates born at high altitudes.

This study aimed to explore the effects of different altitudes on the PPI values of asymptomatic well newborns between 6 and 72 h of life and to obtain reference value ranges of PPI at different altitudes.

## Methods

### Study design and participants

This was a multi-center and prospective cohort study conducted from February 1, 2020, to April 15, 2021. The data of this study were collected from six institutions at different altitudes, which included low altitude (Hainan Women and Children’s Medical Center, 4 m), mild altitude (People’s Hospital of Xinjiang Uygur Autonomous Region, 800 m), moderate altitude (Luchun County People’s Hospital, 1640 m, and Yan’An Hospital affiliated to Kunming Medical University, 1891 m), high altitude (Tibet Autonomous Region People’s Hospital, 3658 m), and higher altitude (Golog Tibetan Autonomous Prefecture People’s Hospital, 4200 m). All infants were recruited consecutively in the neonatal nursery and subsequently received standard postnatal care. The inclusion criteria were infants with an Apgar score ≥ 6 at 5 min of life, no requirement for respiratory support within the first 72 h of life, and clinical and hemodynamic stability. Infants were considered stable according to the following criteria: normal skin color, breathing pattern, posture, tone, and movement, and no apnoeic episodes of greater than 20 s. Newborns who were born extremely preterm and those with congenital malformations (e.g., congenital heart disease, congenital diaphragmatic hernia, or neural tube defects) were excluded from the study. By the time of data analysis, all infants with congenital heart disease found by echocardiography were eliminated.

### PPI measurement

PPI values were measured at 6, 12, 24, 48, and 72 h after birth or before discharge if discharge occurred before 72 h. We used a Masimo Radical-7 motion-resistant pulse oximeter (Masimo, Irvine, CA, USA) and a LNCS Y1 reusable probe secured with multi-site foam wraps. We placed the probes on the infants’ palms and soles. After a stable PPI, the waveform was observed, five consecutive values with a 1-min interval were recorded, and their average value was derived. All measurements were performed by well-trained investigators. The date, time, postnatal age in hours, and the infants’ state (awake or asleep) were recorded accordingly. The data were measured when the infant was sleeping or awake, quiet (no feeding, distress, or crying), and supine. Measurements were obtained on the right palm (RH) and then the sole of the foot with care taken to ensure that the probe was properly positioned to prevent the possibility of optical shunting. Opaque ambient light shields were placed to avoid light exposure while temperature and humidity were adjusted according to birth weight (BW), gestational age (GA), and postnatal age in hours.

### Data collection

Demographic and clinical data were obtained from medical records. The data were then sent to the database based at the Children’s Hospital of Fudan University, Shanghai, China. The purpose-designed database was designed with automatic error-checking systems to ensure accurate data captured from medical records. Before we started the study, the primary investigators at each institution received the same training on how to measure and collect the data. Another trained staff member checked the data regularly, and a data manager carried out weekly spot checks. The clinical data collected included gender, ethnicity, multiparity, GA, birthweight, mode of delivery, Apgar score, and appropriateness for gestational age. Pre- and post-ductal PPI, heart rate, and body temperature were collected at each measurement.

### Definitions

GA was determined based on antenatal ultrasound, menstrual history, obstetric examination, or a combination of all three. If an obstetric evaluation was not available or differed from the postpartum pregnancy evaluation by more than 2 weeks, Ballard scoring was used to evaluate GA [31]. Appropriateness for gestational age was defined as the percentage of birth weight corresponding to GA based on the Chinese reference population, ranging from 10 to 90% [31].

### Statistical analysis

Demographic and anthropometric data of participants were presented as mean and standard deviation or median and interquartile range (IQR) for continuous variables and absolute numbers and percentages for categorical variables. The altitude of each institution was classified as low (0–500 m), mild (500–1500 m), moderate (1500–2500 m), high (2500–4000 m), or higher (> 4000 m) according to the literature review [32].

To assess differences in the PPI values over time at different altitudes, linear mixed-effects models for correlated data were used with adjustment for potential confounders. Highly skewed continuous outcomes (pre- and post-ductal PPI) were first log-transformed before applying regression analyses. Time, altitude groups, and interactions between time and group were included in the models. The potential confounders were gender, ethnicity, multiparity, GA, mode of delivery, Apgar score, appropriateness for gestational age, and factors recorded at each measurement time including heart rate and body temperature. In this model, auto-regressive covariance structures were used for intra-subject correlated errors.

To compare the reference range of PPI at different altitudes over different postnatal ages, 50th percentile curves for each altitude at 6 to 72 h of life were fitted using Cole’s Lambda Mu Sigma (LMS) method [33]. The LMS method was conducted using R version 3.6.1 with the GAMLSS package. The LMS approach initially estimates the three parameters of the Box-Cox transformation of the distribution of the measurement. The L determines a nonlinear conversion of PPI, such that its distribution approximates the normal distribution. The M stands for the mean of that normal distribution, and the S represents the coefficient of variation. The three parameters are constrained to change smoothly as the covariate changes [34]. L, M, and S correspond to the following formulas: *Z* = (X/M)L 1/LS, where X indicates the measured value of PPI and the centile = M* (1 + L* S* Zα)1/L, where Zα is the z-score that corresponds to the given percentile. The Z-score measures the distance in SDs of a sample from the mean [35]. All data management and statistical analyses were performed using SAS version 9.4 (SAS Institute, Cary, North Carolina), and R version 3.6.1. A 2-sided *P* value of 0.05 was used to determine the statistical significance.

## Results

### Study population

**Fig. 1:**
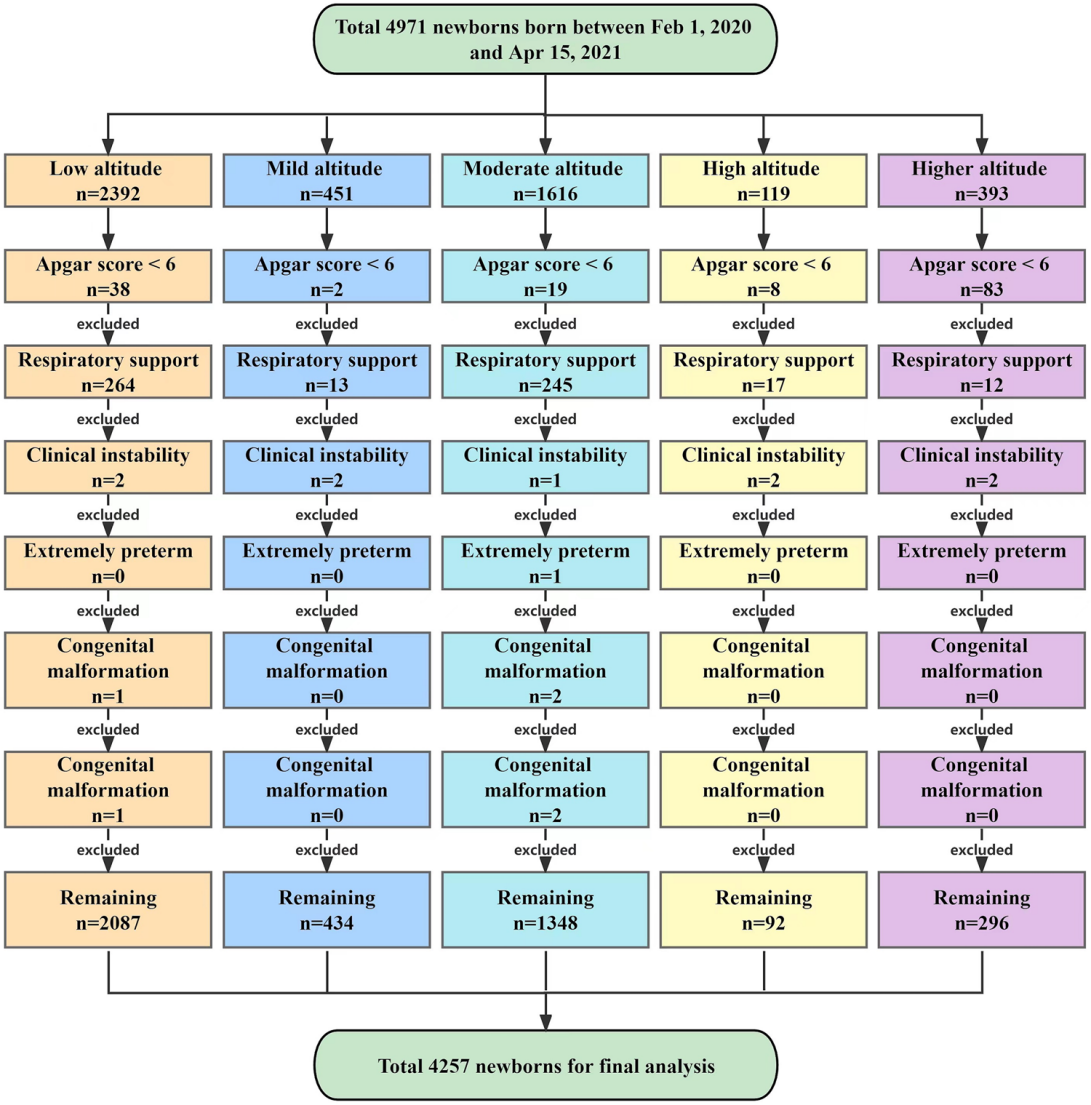
Inclusion and exclusion flow chart

A total of 4971 infants were delivered at the six institutions during the study period, of whom 4257 were eligible and included in the final analysis (Fig. 1). Among them, 2339 (54.9%) were boys; 2615 cases (61.4%) were ethnic Han; 3988 cases (93.7%) were singletons; 2551 cases (59.9%) were delivered vaginally, and 1706 cases (40.1%) were born by cesarean section. The medians of GA and BW were 39.00 (38.00, 39.90) weeks and 3150 (2800, 3450) grams, respectively. The median and quartile pre- and post-ductal PPI at different altitudes were 1.70 (1.20, 2.60) and 1.70 (1.10, 2.70) for all infants, 1.30 (1.10, 1.90) and 1.10 (0.88, 1.80) at low altitude, 1.40 (1.00, 2.00) and 1.30 (0.99, 2.00) at mild altitude, 1.90 (1.30, 2.50) and 1.80 (1.20, 2.70) at moderate altitude, 1.80 (1.40, 3.50) and 2.20 (1.60, 4.30) at high altitude, and 3.20 (2.70, 3.70) and 3.10 (2.10, 3.30) at higher altitude, respectively. The demographic characteristics and measurement results of the study population were presented in Table 1.

**Table 1:** Demographic data and measurement results

**Characteristics**	**Low altitude**	**Mild altitude**	**Moderate altitude**	**High altitude**	**Higher altitude**	**Total**
	*n* = 2087	*n* = 434	*n* = 1348	*n* = 92	*n* = 296	*n* = 4257
Males, n (%)	1183 (56.68%)	235 (54.15%)	715 (53.04%)	48 (52.17%)	158 (53.38%)	2339 (54.94%)
Han ethnic group, n (%)	2033 (97.41%)	253 (58.29%)	329 (24.42%)	0 (0%)	0 (0%)	2615 (61.44%)
Singleton birth, n (%)	1973 (94.54%)	399 (91.94%)	1258 (93.32%)	87 (94.57%)	271 (91.55%)	3988 (93.68%)
Gestational age, median (IQR), week	39.00 (38.10, 39.90)	38.90 (38.10, 39.70)	39.00 (37.40, 40.00)	38.00 (37.00, 39.40)	38.00 (37.00, 39.00)	39.00 (38.00, 39.90)
Birth weight, median (IQR), g	3150 (2900, 3450)	3400 (3120, 3750)	3100 (2700, 3400)	2952.5 (2502.5, 3255)	3000 (2725, 3250)	3150 (2800, 3450)
Vaginal delivery, n (%)	1090 (52.23%)	136 (31.34%)	982 (72.85%)	63 (68.48%)	280 (94.59%)	2551 (59.92%)
Cesarean section, n (%)	997 (47.77%)	298 (68.66%)	366 (27.15%)	29 (31.52%)	16 (5.41%)	1706 (40.08%)
5-min Apgar score, median (IQR)	10.00 (10.00, 10.00)	10.00 (10.00, 10.00)	9.00 (9.00, 10.00)	10.00 (10.00, 10.00)	8.00 (8.00, 9.00)	10.00 (9.00, 10.00)
Appropriateness for gestational age, n (%)	1894 (90.75%)	390 (89.86%)	1086 (80.56%)	73 (79.35%)	239 (80.74%)	3682 (86.49%)
Pre-ductal PPI, median (IQR)	1.30 (1.10, 1.90)	1.40 (1.00, 2.00)	1.90 (1.30, 2.50)	1.80 (1.40, 3.50)	3.20 (2.70, 3.70)	1.70 (1.20, 2.60)
Post-ductal PPI, median (IQR)	1.10 (0.88, 1.80)	1.30 (0.99, 2.00)	1.80 (1.20, 2.70)	2.20 (1.60, 4.30)	3.10 (2.10, 3.30)	1.70 (1.10, 2.70)

### The influence of altitude on PPI values at different measurement time points

**Fig. 2:**
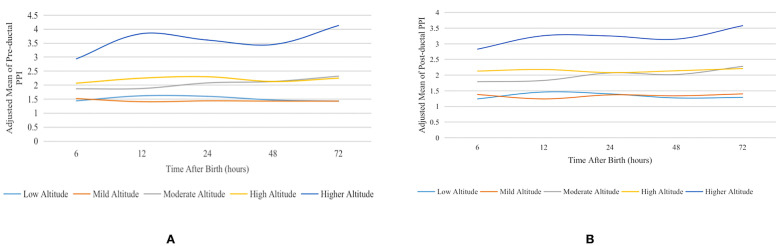
Adjusted mean curves of PPI at different altitudes at different times after birth

Linear mixed-effects regression was performed to investigate the association between PPI values and different altitudes with adjustment for possible covariates, including gender, ethnicity, multiple pregnancies, GA, Cesarean delivery, Apgar score, appropriateness for gestational age, and the factors at each measurement time point including heart rate and body temperature. The adjusted mean and 95% Cl of pre- and post-ductal PPI at different altitudes at different measurement time points are shown in Table 2, and the curves of this are shown in Fig. 2.

**Table 2:** Adjusted mean of PPI at different altitudes at different times after birth

**PPI**	**Time after birth (hours)**	Adjusted mean (95% CI) at different altitude groups*				
		**Low altitude**	**Mild altitude**	**Moderate altitude**	**High altitude**	**Higher altitude**
Pre-ductal PPI	6	1.44 (1.31, 1.58)	1.52 (1.38, 1.67)	1.87 (1.71, 2.04)	2.07 (1.80, 2.38)	2.94 (2.65, 3.26)
	12	1.62 (1.48, 1.77)	1.41 (1.28, 1.56)	1.88 (1.72, 2.05)	2.25 (1.95, 2.59)	3.84 (3.47, 4.26)
	24	1.60 (1.46, 1.76)	1.44 (1.31, 1.59)	2.08 (1.91, 2.27)	2.30 (1.97, 2.70)	3.61 (3.25, 4.01)
	48	1.47 (1.34, 1.62)	1.43 (1.30, 1.57)	2.13 (1.94, 2.34)	2.13 (1.74, 2.61)	3.45 (3.10, 3.83)
	72	1.43 (1.30, 1.57)	1.43 (1.29, 1.57)	2.32 (2.11, 2.55)	2.25 (1.75, 2.90)	4.13 (3.70, 4.62)
Post-ductal PPI	6	1.24 (1.12, 1.37)	1.38 (1.25, 1.52)	1.79 (1.63, 1.96)	2.13 (1.84, 2.47)	2.83 (2.54, 3.16)
	12	1.46 (1.33, 1.60)	1.24 (1.12, 1.37)	1.83 (1.67, 2.01)	2.18 (1.88, 2.53)	3.26 (2.93, 3.64)
	24	1.40 (1.27, 1.55)	1.37 (1.24, 1.52)	2.07 (1.89, 2.26)	2.08 (1.77, 2.46)	3.25 (2.91, 3.63)
	48	1.27 (1.15, 1.41)	1.34 (1.22, 1.49)	2.02 (1.84, 2.23)	2.14 (1.73, 2.64)	3.15 (2.82, 3.52)
	72	1.29 (1.16, 1.42)	1.40 (1.26, 1.55)	2.28 (2.07, 2.52)	2.21 (1.71, 2.86)	3.58 (3.18, 4.02)

*PPI*, peripheral perfusion index; *CI*, confidence interval

*Adjusted for gender, ethnicity, multiparity, gestational age, cesarean delivery, 5-min Apgar score, appropriateness for gestational age, heart rate at each measurement time point, and body temperature at each measurement time point

**Fig. 3:**
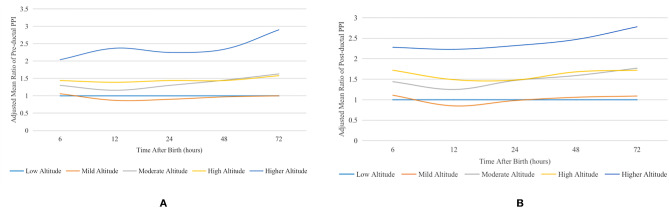
Adjusted mean ratio curves of PPI at different altitudes at different times after birth

Overall, the adjusted means of pre- and post-ductal PPI increased with altitudes. Using the group at low altitude as a reference, the adjusted mean ratio and 95% Cl of pre- and post-ductal PPI at different altitudes at different measurement times are shown in Table 3, and the curves of this are shown in Fig. 3. For pre- and post-ductal PPI, the values generally increased with the increase in altitude, especially at the higher altitude range. The change of PPI values at mild altitude may be due to the small sample size at this altitude, especially compared with those at low and moderate altitudes.

**Table 3:** Adjusted mean ratio of PPI at different altitudes at different times after birth

**PPI**	**Time after birth (months)**	Adjusted mean ratio (95% CI) at different altitudes*				
		**Low altitude**	**Mild altitude**	**Moderate altitude**	**High altitude**	**Higher altitude**
Pre-ductal PPI	6	*Ref*	1.06 (0.99, 1.13)	1.30 (1.22, 1.37)	1.44 (1.27, 1.63)	2.04 (1.88, 2.22)
	12	*Ref*	0.87 (0.82, 0.92)	1.16 (1.11, 1.22)	1.39 (1.23, 1.57)	2.37 (2.19, 2.56)
	24	*Ref*	0.90 (0.84, 0.96)	1.30 (1.23, 1.37)	1.44 (1.24, 1.66)	2.25 (2.07, 2.45)
	48	*Ref*	0.97 (0.91, 1.04)	1.45 (1.35, 1.55)	1.44 (1.19, 1.75)	2.34 (2.14, 2.56)
	72	*Ref*	1.00 (0.93, 1.08)	1.63 (1.52, 1.75)	1.58 (1.24, 2.02)	2.90 (2.63, 3.19)
Post-ductal PPI	6	*Ref*	1.11 (1.03, 1.19)	1.44 (1.35, 1.53)	1.72 (1.50, 1.96)	2.28 (2.08, 2.49)
	12	*Ref*	0.85 (0.80, 0.90)	1.25 (1.19, 1.32)	1.49 (1.31, 1.70)	2.23 (2.06, 2.43)
	24	*Ref*	0.98 (0.92, 1.05)	1.47 (1.39, 1.56)	1.48 (1.27, 1.73)	2.32 (2.12, 2.53)
	48	*Ref*	1.06 (0.98, 1.14)	1.59 (1.48, 1.71)	1.68 (1.38, 2.06)	2.47 (2.25, 2.71)
	72	*Ref*	1.09 (1.01, 1.18)	1.77 (1.65, 1.91)	1.72 (1.34, 2.21)	2.78 (2.51, 3.07)

*PPI*, peripheral perfusion index; *CI*, confidence interval

*Adjusted for gender, ethnicity, multiparity, gestational age, cesarean delivery, 5-min Apgar score, appropriateness for gestational age, heart rate at each measurement time point, and body temperature at each measurement time point

### Smoothed reference curves at the 50th percentile for pre- and post-ductal PPI values at different altitudes

**Fig. 4:**
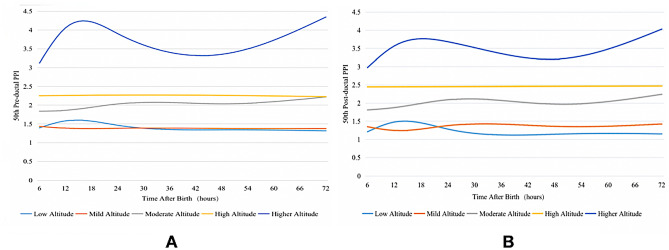
Smoothed reference curves for PPI values at the 50th percentile of different altitudes

Smoothed reference curves for pre- and post-ductal PPI values at the 50th percentile of different altitudes are shown in Fig. 4. With the exception of those at mild altitude, both pre- and post-ductal PPI increased with altitude, and PPI values at mild and low altitude were similar. The PPI curves at low, mild, moderate, and high altitudes were generally stable with small fluctuations over time, but the PPI curves at higher altitude fluctuated greatly. In addition, as shown in Table 4, the 50th percentile curves of pre- and post-ductal PPI values for newborns at mild, low, moderate, and high altitudes were relatively close to each other, while the 50th percentile curve at the higher altitude was noticeably further from those at other altitudes. With the exception of higher altitude, the values of PPI at other altitudes changed little with time. At each time point, the values increased with the increase in altitude.

**Table 4:** The 50th percentile PPI at different altitudes at different times after birth

**PPI**	**Time after birth (months)**	**Low altitude**	**Mild altitude**	**Moderate altitude**	**High altitude**	**Higher altitude**
Pre-ductal PPI	6	1.40	1.43	1.84	2.25	3.12
	12	1.58	1.38	1.86	2.26	4.06
	24	1.46	1.38	2.03	2.27	3.90
	48	1.34	1.38	2.04	2.26	3.35
	72	1.31	1.37	2.22	2.23	4.35
Post-ductal PPI	6	1.21	1.35	1.81	2.45	2.98
	12	1.48	1.25	1.88	2.45	3.57
	24	1.28	1.38	2.09	2.45	3.68
	48	1.14	1.36	1.97	2.46	3.20
	72	1.15	1.43	2.24	2.47	4.03

*PPI*, peripheral perfusion index

## Discussion

This was one of the largest studies reporting the reference ranges of neonatal PPI at different altitudes. Our study demonstrated that the PPI of infants in areas above sea level was relatively higher. We included infants who were relatively stable, so their blood pressure was relatively stable. The median and quartile of pre- and post-ductal PPI showed that these values increased with the increase of altitude, which may be due to the decrease of atmospheric pressure, vasodilation, and high blood flow with the increase of altitude. At relatively high altitude areas, as the atmosphere pressure decreases, the oxygen pressure also decreases, and the oxygen entering the alveoli through the airway decreases. Oxygen consumption is related to cardiac output, heart rate, hemoglobin levels, and the oxygen-carrying capacity of hemoglobin. Oxygen demand is constant, and the body compensates by adjusting cardiac output. As mentioned above, from the perspective of PPI detection principles, cardiac output is the most direct factor affecting PPI. The reason for the great distance between the curve at the higher altitude and the other four altitudes may be that the oxygen at the higher altitude is extremely thin. In this case, the heart needs to pump more blood to maintain the oxygen consumption of the body, thereby increasing the values of PPI dramatically. There have been many studies [5, 7, 11, 36] on PPI values in the past, but there is a lack of research on the influence of different altitudes on PPI values. A study [37] investigated the pre- and post-ductal PPI values of newborns with GA more than 35 weeks during 24–48 h after birth at an altitude of 1790 m. The median pre- and post-ductal PPI were 2.10 and 2.20, respectively. The pre-ductal PPI values were almost the same as our results while the post-ductal PPI values were slightly different from our study at the same altitude area within the same period of life. Our study also described significant changes in PPI trends in infants at different altitudes at 6 to 72 h of life. This may reflect the physiological changes of peripheral vascular blood flow after birth and may be related to the transition from fetal circulation to normal circulation in the first few days of life.

Our study demonstrates that PPI values are different at different altitudes. We provided a constant range of PPI in the early postnatal period of asymptomatic well neonates at different altitudes, which can be used for the identification of neonates with abnormal perfusion in clinical practice. Furthermore, the corresponding disease screening PPI thresholds should also be modified for different altitudes. In the current study, we excluded infants with CCHD and found that the PPI values of all the infants included were more than 0.7, which was not inconsistent with the result in the previous study [10]. We will continue our study to make out the PPI threshold value in screening neonatal CCHD at different altitudes.

Our study has limitations. The sample size at each altitude was unbalanced. This may be the reason why the rule that PPI values increase with altitudes is not applicable at some time points at mild altitude. However, it does not affect the overall trend. Our study only focused on the PPI of healthy, asymptomatic infants within 72 h of birth, and the observation time was limited. The trend of PPI over a longer period after birth was not explored.

Our study monitored the health status of infants at enrollment and during the study period through clinical assessment, respiratory and cardiovascular parameter measurements, and blood analysis. In addition, very preterm infants were not recruited to this study because they tended to be particularly unstable when undergoing invasive procedures (e.g., mechanical ventilation and CVC) that may affect PPI. Pathological conditions, such as congenital heart disease with low blood volume and left-to-right shunting, often occur in the neonatal transition period, which changes early vascular blood flow and cardiovascular adaptation [38–40]. To eliminate these factors that might affect PPI, we excluded those infants in this study. The relationship between changes in PPI values and specific diseases deserves further study, and we will try to explore this in the future.

## Conclusions

With the increase of altitude, pre- and post-ductal PPI of newborns increases. Our study obtained the PPI reference values of asymptomatic well newborns at 6 to 72 h after birth at different altitudes from 4 to ≥ 4000.

## Data Availability

The data that support the findings of this study are available from the corresponding author upon reasonable request.
